# Therapeutic Effectiveness of Lumbar Medial Branch Block and Lumbar Intra-Articular Facet Injections: A Propensity-Matched Cohort Study

**DOI:** 10.7759/cureus.79962

**Published:** 2025-03-03

**Authors:** Ian S Ackers, Jacquelyn A Witzke, Arvin Saremi, Tyler K Farley, Nicolas R Thompson, Yadi Li, Brian D Foresi, Kush K Goyal

**Affiliations:** 1 Physical Medicine and Rehabilitation, Michigan State University, East Lansing, USA; 2 Physical Medicine and Rehabilitation, Mary Free Bed Rehabilitation Hospital, Grand Rapids, USA; 3 Radiology, University of Massachusetts Chan Medical School, Worcester, USA; 4 Physical Medicine and Rehabilitation, Charleston Area Medical Center, Charleston, USA; 5 Quantitative Health Sciences, Cleveland Clinic Foundation, Cleveland, USA; 6 General Surgery, OhioHealth Riverside Methodist Hospital, Columbus, USA; 7 Center for Spine Health, Cleveland Clinic Foundation, Cleveland, USA

**Keywords:** chronic low back pain, facet injection, intra-articular facet joint injection, low back pain, medial branch block

## Abstract

Background: Chronic low back pain is a prevalent condition that is treated commonly with radiofrequency neurotomy (RFN) after diagnostic targeting with medial branch blocks (MBB) or intra-articular (IA) injection.

Purpose: We evaluated the long-term therapeutic value of MBB and IA injection of steroids for relief from chronic low back pain beyond the current diagnostic utility.

Methods and outcome measures: This was a retrospective propensity-matched cohort study from a single physician injection census at a tertiary care hospital. A total of 460 patients receiving MBB (n=383) or IA injection (n=77) in 2013-2020 were included. Primary outcome measures were patient-reported outcomes (PROs) at the time of injection, and follow-up at three and six months with the Numerical Rating Scale (NRS), PRO Measurement Information System (PROMIS)-Mental Health (MH), PROMIS-Physical Health (PH), and the Patient Health Questionnaire (PHQ)-9 scores. The same PROs at the one-year follow-up were the secondary outcome measures. Propensity weighting was performed to balance MBB and IA injection groups over several demographic and clinical categories.

Results: Significant improvements in NRS (p=<0.001) were reported at the three-month, six-month, and one-year follow-ups, while significant improvements in PROMIS-PH scores (p=0.015) were identified at three and six months post injection.

Conclusions: Our results suggest that MBB and IA injection have potential therapeutic benefits for chronic low back pain for at least six months post injection. These results suggest that there is value in these diagnostic modalities therapeutically beyond the acute time frame. These results lay the groundwork for additional investigations into treatment options for patients affected by chronic low back pain.

## Introduction

Lumbar facet joint (LFJ) pain is a common cause of low back pain (LBP). The annual prevalence of LBP in the American population ranges from 15% to 45% and with a lifetime incidence as high as 80%, LBP continues to be the leading cause of years lived with disability in developed countries [1-3]. The facet joints have become an important therapeutic target, as these joints are common contributors to LBP. The LFJs are small joints located between spinal vertebrae and when these joints become inflamed or injured can cause pain and discomfort that may be acute, chronic, or recurrent. The diagnosis and treatment of facetogenic LBP is difficult, with no established pathognomonic historical, physical exam, or imaging findings that reliably predict spine pain generators [4,5]. If conservative management yields insufficient relief, interventional diagnostic/therapeutic procedures may be considered, targeting the lumbar spinal segment, i.e. LFJs and intervertebral discs.

The paradigm of two test injections followed by therapeutic radiofrequency neurotomy (RFN) is currently the recommended pathway for diagnosis and treatment of axial and facetogenic LBP, however, the number of test injections and the threshold of pain relief is controversial [6-8]. RFN is a minimally invasive procedure that uses heat to destroy the nerve fibers that transmit afferent pain signals from the facet joints. Recent studies have shown a significant increase in the utilization of RFN for LFJ pain, with an overall increase of 130% between 2007 and 2016 [9]. Before undergoing RFN, diagnostic injections such as intra-articular (IA) facet joint injections and medial branch blocks (MBB) are often used to confirm the diagnosis and predict the outcome of future RFN [10].

The IA injection involves the injection of an anti-inflammatory medication and an anesthetic into the joint capsule of the facet joint to reduce inflammation and pain. The effectiveness of IA steroid injection into the LFJ is not clear [11,12]. MBB also targets the LFJ and involves an injection of a local anesthetic around the medial nerves that supply respective facet joints above and below the lesion, thereby temporarily blocking the transmission of pain signals. MBBs are less technically difficult to perform than IA injections as degenerative arthritis may increase the difficulty in accessing the posterior IA joint [13]. Hardware obstruction and facet arthropathy may also limit the ability to reliably access the junction of the lateral aspect of the superior articular process and transverse process in MBBs. This highlights the need for flexibility for the proceduralist to adapt to each patient’s underlying anatomy or clinical limitations to maximize outcomes.

The effectiveness of IA injection vs MBB vs RFN is debated, but at least one scoping review suggests that MBB has a higher level of evidence (Level II-1 to II-2) of effectiveness compared to RFN (Level II-2 to II-3) based on the United States Preventive Services Task Force level of evidence criteria [14]. Further, the cost-effectiveness of MBBs prior to RFN has not been evaluated clearly [8]. Therapeutic MBB has been investigated in multiple studies and found to provide significant improvement in functional and pain outcomes related to LBP with documented benefit at two years post injection [15,16]. Presumably, the addition of steroids should facilitate the reduction of medial branch nerve irritation secondary to inflammation. However, the exact mechanism for long-term relief of LBP after block is unclear.

Currently, MBB and IA injections are utilized to predict the effectiveness of RFN. However, there is evidence that these injections may also provide long-term therapeutic benefits for LFJ pain [17,18]. To date, the literature has not fully clarified the extent of the therapeutic potential of MBB and IA injection for the long-term management of chronic LBP.

We hypothesized that patients who receive fluoroscopically guided lumbar MBB and IA injection with local anesthetic and steroids for IA-mediated pain experience a clinically significant reduction in pain levels, as well as improvement in function and psychosocial parameters affected by chronic LBP that persists. We hypothesized that, despite current literature not supporting the therapeutic utility of these injections, patients may experience worthwhile improvement of symptoms extending beyond the timeframe observed during diagnostic injections.

## Materials and methods

This was a retrospective observational propensity-matched cohort study of patients receiving either lumbar MBB or IA injection (Current Procedural Terminology code (64493) and (64494), respectively [19]) between September 2013 and March 2020. The study was approved by the Institutional Review Board of the Cleveland Clinic Foundation (approval number: 21-631). Verbal and written informed consent for study inclusion was obtained from each patient.

Inclusion and exclusion criteria

Patients with suspected LFJ pain based on history, examination, and imaging findings who had been treated by either an MBB or IA injection from a single physician provider were included. The procedures were performed by a fellowship-trained Physical Medicine & Rehabilitation staff physician in the Center for Spine Health, who completed a Spine Medicine Fellowship at the Cleveland Clinic. Patients were classified into either the MBB or IA injection group by the type of their first injection. Patients with multiple same-day injections were excluded. In addition, patients were assessed preoperatively to determine a possible diagnosis other than LFJ dysfunction. If the patient was determined to have a different etiology/diagnosis for their pain, they were excluded from the study. All eligible patients who fulfilled the inclusion/exclusion criteria during the study period were included.

Data collection

In 2015, our institution replaced a previously established patient-reported questionnaire (EuroQol five-dimensional questionnaire (EQ-5D)) with the Patient-Reported Outcomes Measurement Information System (PROMIS) Global Health scale. Patient characteristics and patient-reported outcome (PRO) scores of the Numerical Rating Scale (NRS) [20], PROMIS-Mental Health (PROMIS-MH), PROMIS-Physical Health (PROMIS-PH) [21], and the Patient Health Questionnaire (PHQ-9) [22] were summarized by mean with standard deviation for continuous variables, and count with percentage for categorical variables.

The primary outcomes for this study were PRO scores at three and six months (0-120 and 121-240 days, respectively) after the injection. Secondary outcomes were PRO scores at pre-injection, one year (241-540 days after injection), and two years (541-900 days after injection, respectively). Very few IA and MBB patients had NRS at the two-year follow-up, so the two-year time point was not included in MBB and IA analyses. 

Only data from their first injection were used in analyses. For patients who had both MBB and IA injections, PRO scores were excluded if measured after a second type of injection.

Procedure description

After obtaining both verbal and written informed consent, each patient was placed in a prone position on the fluoroscopic table in the procedure room, and the posterior lumbosacral spine was prepped and draped in the usual sterile fashion using iodine or chlorhexidine. Local anesthesia was achieved with superficial skin wheal(s) using 1% preservative-free lidocaine near the junction of the targeted transverse and superior articular processes and/or sacral ala which was localized by counting from the intersection of the iliac crest. Through the skin wheal, a 22-gauge, 3.5-inch (or 22-gauge, 5-inch needle for patients with larger body habitus) curved Quincke-tip spinal needle was used.

For MBB injections, the spinal needle was advanced under direct fluoroscopic visualization in the anteroposterior (AP), ipsilateral oblique, and lateral planes, until the needle tip reached a position perpendicular to the mammillary processes at the vertebral body and/or sacral ala. For IA injections, the spinal needle was advanced under direct fluoroscopic visualization in the AP, ipsilateral oblique, and lateral planes, until the needle tip reached the intra-articular joint space.

**Figure 1:**
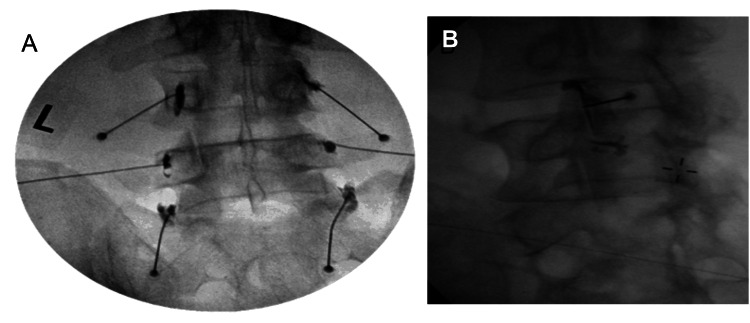
(A) Representative MBB injection. Anteroposterior view of the lumbar spine with needle placement and contrast flow, confirming appropriate location for MBB; (B) Representative IA facet joint injection. Oblique view of the lumbar spine with needle placement within the facet joint, confirmed by contrast flow outlining the joint both superiorly and inferiorly. MBB: Medial Branch Block; IA: Intra-articular

For both procedures, proper needle placement was confirmed using 0.1-0.2 ml of OMNIPAQUE™ 180 nonionic confirming contrast (GE Healthcare, Chicago, Illinois, United States) at each target without any intravascular uptake of contrast seen under direct fluoroscopic visualization in the AP, oblique, and lateral planes (Figure 1). Adequate hemostasis was obtained at the needle puncture site. The injection site was cleaned, and a sterile dressing was applied.

Patients received 1 cc of 80 mg Depo-Medrol with 0.75% Marcaine divided evenly. In a six-needle injection, bilateral L3, L4 MBB, and L5 dorsal ramus were treated with approximately 13.3 mg of Depo-Medrol at each location, along with 0.3 cc of 0.75% Marcaine. In a three-needle injection, unilateral L3, L4 medial branch, and L5 dorsal ramus were treated with approximately 26.6 mg of Depo-Medrol at each location, along with 0.3 cc of 0.75% Marcaine. In an MBB injection, a total of 0.3-0.4 ml of injectate was administered per medial branch or dorsal rami nerve.

Data analysis

Propensity score weighting was used to achieve a covariate balance between the two treatment groups. Propensity scores were estimated using generalized boosted models (GBM) utilizing the twang package in R (R Foundation for Statistical Computing, Vienna, Austria) [23,24]. In propensity score estimation, the dependent variable was the treatment groups (MBB, IA injection) and predictors included demographics and baseline characteristics. To assess covariate balance between groups, we computed mean ± standard deviation (SD) for continuous variables and proportion ± SD for categorical variables within each group. Balance was assessed using standardized bias (i.e., absolute standardized mean difference), which is the absolute value of the difference in means (proportions) between the two groups divided by the SD in the full sample. Balance before and after propensity score weighting was assessed by raw and weighted group means (proportions), respectively. Covariates with a standardized bias greater than 0.2 were considered imbalanced.

The proportion of patients exceeding minimal clinically important difference (MCID) for each outcome was computed. A total point change of >2.5 for NRS and ≥ 5 for PHQ-9 was considered clinically significant [25-27]. The same approach was used to determine an MCID of 5 for both PROMIS-MH and PROMIS-PH. This is an established method for the interpretation of changes in health-related quality-of-life measures. For NRS, the frequency and percentage of patients with improvement by 50% (e.g., if baseline NRS score was 8, then a follow-up score of 4 or less was considered as 50% improvement) was reported.

Mixed-effect linear regression models were used to model the PROs at five time points (pre-injection and three-month, six-month, one-year, and two-year post-injection follow-ups). Fixed-effect independent variables included the treatment group (MBB, IA injection), time point, and the interaction between the treatment group and time point. Demographic and clinical variables that were imbalanced after propensity score weighting were included in the models as covariates except for PROMIS-MH, PROMIS-PH, and PHQ-9 at pre-injection, which had missing data. These covariates were treated as fixed effects. A random effect for patients was also included. Weights from propensity score estimation were incorporated into the models. All tests were two-sided, and the significance was set at 0.05.

## Results

A total of 460 patients were enrolled in the study, of which 383 underwent MBB and 77 received IA. Both the MBB and IA injection groups had similar proportions of female participants at 54.8% and 53.2%, respectively. The mean age of all study participants at the time of injection was 56.57 years (MBB, 56.84 ± 15.38 years; IA injection, 56.30 ± 15.42 years). The average BMI for all participants was 31.4 kg/m^2^ (MBB, 31.36 ± 7.51 kg/m^2^; IA injection, 29.51 ± 6.38 kg/m^2^) (Table 1). A summary of all subject assessments for PRO at three months, six months, and one year are reported in Table 2.

**Table 1:** Characteristics of participants (N=460) Data presented as frequency (percentage) except in age, which is given as mean±SD BMI: Body Mass Index; PT: Physical Therapy; RFN: Radiofrequency Neurotomy; SI: Sacroiliac

Characteristic	Frequency (Percentage)
Age at Injection (years), mean±SD	56.3±15.5
Female	248 (54.6)
BMI	460 (100)
Race	
White	301 (65.4)
African American	69 (15.0)
Other	90 (19.6)
Type of Lumbar Injection	
Intra-articular	77 (16.7)
Medial branch block	383 (83.3)
Side	459 (99.8)
Right	11 (2.4)
Left	11 (2.4)
Bilateral	438 (2.4)
Steroid used	
Kenalog (40mg)	40 (0.88)
Kenalog (80mg)	20 (0.44)
Depomedrol (40mg)	12 (2.6)
Depomedrol (80mg)	425 (93.0)
Celestone	12 (2.6)
History	
Active Smoker	104 (22.7)
History of PT	352(77.7)
History of Lumbar Surgery	69 (15.0)
History of Lumbar Fusion	34 (7.4)
History of Remote Lumbar Facet Injection	81 (17.7)
History of Lumbar Facet RFA	25 (5.4)
History of SI Joint Injection ± 2 years	97 (21.1)
Spondylolisthesis	116 (25.3)
Scoliosis	
Degenerative	147 (32)
Idiopathic	19 (4.1)

**Table 2:** Subject assessments PRO: Patient Reported Outcome; NRS: Numeric Rating Scale; PROMIS: Patient-Reported Outcomes Measurement Information System, PHQ-9: Patient Health Questionnaire-9

Assessment	Number of participants	Score, mean (SD)
PRO Baseline		
NRS	458	7.1 (1.7)
PROMIS Mental Health	256	42.6 (9.8)
PROMIS Physical Health	254	36.3 (6.5)
PHQ-9	273	8.8 (6.7)
PRO 3 Months		
NRS	174	2.4 (2.1)
PROMIS Mental Health	188	43.5 (9.4)
PROMIS Physical Health	186	38.4 (7.3)
PHQ-9	213	7.2 (6.0)
PRO 6 Months		
NRS	34	2.2 (2.4)
PROMIS Mental Health	123	42.6 (10.2)
PROMIS Physical Health	124	37.8 (7.3)
PHQ-9	143	8.1 (6.4)
PRO 1 Year		
NRS	21	2.0 (1.9)
PROMIS Mental Health	156	42.9 (9.9)
PROMIS Physical Health	155	37.8 (7.4)
PHQ-9	148	8.1 (6.5)

We were unable to directly compare MBB or IA in the analysis due to study design and insufficient power. Before propensity score weighting, 10 variables were imbalanced between the two groups: age, BMI, side, steroid, history of physical therapy, spondylolisthesis, and PROs at pre-injection (NRS, PROMIS-MH, PROMIS-PH, PHQ-9). Patients in the MBB group were older, had higher BMI, higher (worse) NRS and PHQ-9 scores at pre-injection, lower (worse) PROMIS-MH and PROMIS-PH scores at pre-injection, more likely to have bilateral injection and history of physical therapy, and less likely to have spondylolisthesis.

**Figure 2:**
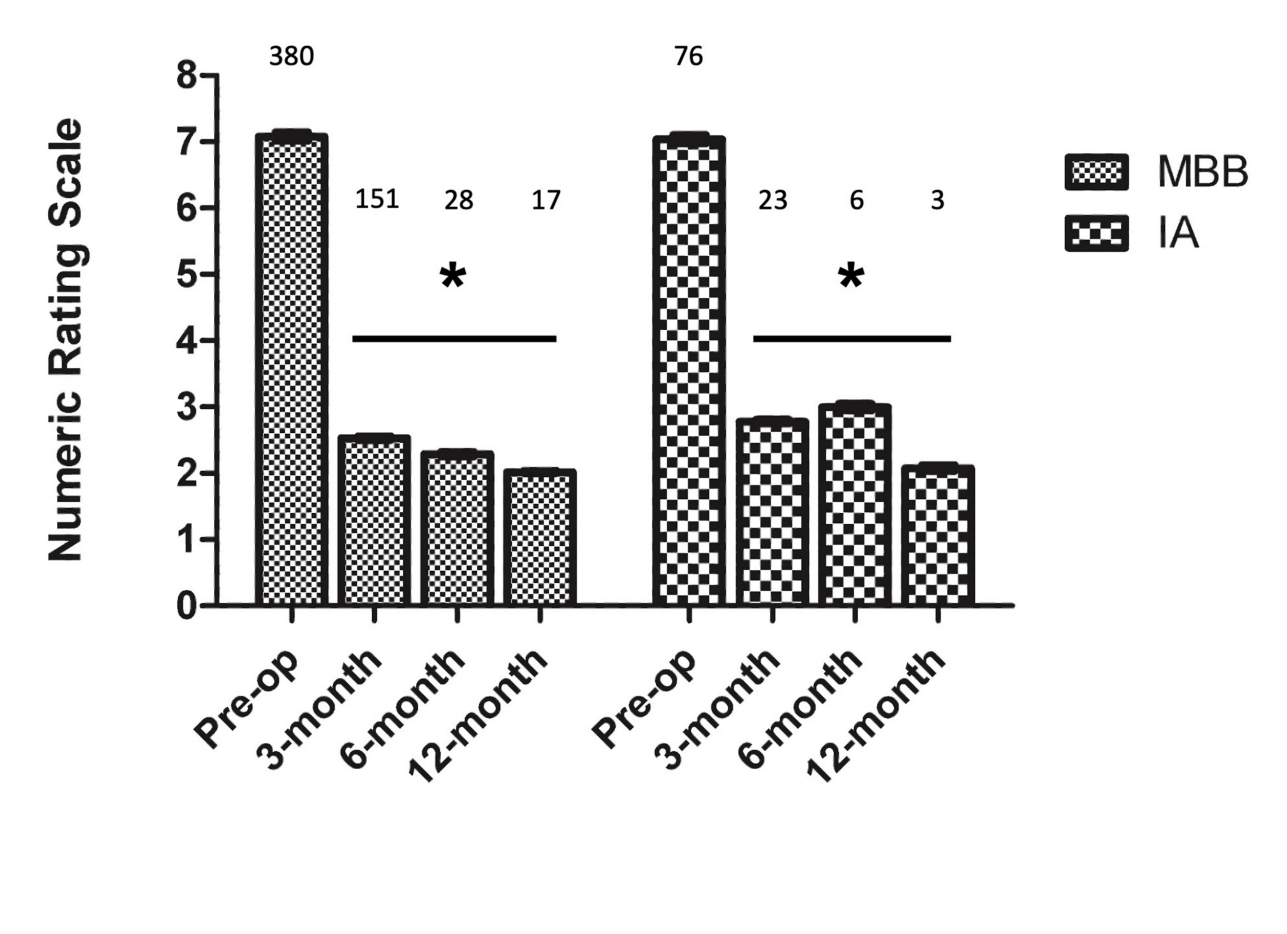
NRS scores before and after injection Absolute number of participants is indicated at each time point. * = p<0.05 NRS: Numerical Rating Scale; IA: Intra-articular; MBB: Medial Branch Block

When examining the patients’ improvement in NRS pain scores, there was a statistically significant reduction of pain in both MBB and IA injection groups at follow-up time points of three months, six months, and one year (Figure 2).

The interaction between groups and time points was not significant (P = 0.273). The trajectory of NRS scores across time did not significantly differ between the two groups. The mean NRS score assessed pre-operatively was 7.0 in the IA injection group and 7.1 in the MBB group. The reduction in NRS score was -4.25, -4.03, and -4.96 for IA injection and -4.55, -4.80, and -5.06 for MBB at the three-month, six-month, and one-year follow-up, respectively. MCID for pain scores is generally considered a change in NRS of 2.5 [27]. In our study, patients achieved MCID on average for both groups and experienced approximately 55-70% pain relief from baseline at these time points.

The number of patients who followed up at three-, six-, and 12-months decreased over the study period (N=389, N=66; N=48, respectively). Follow-up for each group (MBB, IA) is presented in Table 3. In patients who were followed up, the frequency and percentage of patients who improved by MCID demonstrated a strong response (greater than 85% improvement) to either MBB or IA injection at the three-month, six-month, and one-year follow-up (Table 3). Additionally, for NRS, the frequency and percentage of patients with improvement by at least 50% (e.g., if the baseline NRS score was 8, then a follow-up score of 4 or less was considered as 50% improvement) is also presented in Table 3. Further, the frequency and percentage of patients whose NRS scores improved by 50%, 75%, 90%, and 100% are reported in Table 4.

**Table 3:** Frequency and percentage of patients who improved by MCID and by 50% in each group. NRS: Numerical Rating Scale; MCID: Minimal Clinically Important Difference

Change from baseline to follow-up	Medial Branch Block (n=383)		Intra-articular Injecion (n=77)	
	Number of participants	Patients who showed improvement, n (%)	Number of participants	Patients who showed improvement, n (%)
Improvement by MCID				
NRS				
3-month	151	116 (76.8)	23	19 (82.6)
6-month	28	24 (85.7)	6	4 (66.7)
1-year	18	16 (88.9)	3	2 (66.7)
Improvement by 50%				
NRS				
3-month	151	114 (75.5)	23	20 (87.0)
6-month	28	22 (78.6)	6	5 (83.3)
1-year	18	16 (88.9)	3	2 (66.7)

**Table 4:** Pain relief after injection (medial branch block or intra-articular injection) at each follow-up interval

Time Point	Number of participants	50% Relief, n (%)	75% Relief, n (%)	90% Relief, n (%)	100% Relief, n (%)
3-month follow-up	389	285 (73.3%)	181 (46.5%)	100 (25.7%)	52 (13.4%)
6-month follow-up	66	58 (87.9%)	39 (59.1%)	24 (36.4%)	10 (15.2%)
12-month follow-up	48	41 (85.4%)	32 (66.7%)	23 (47.9%)	12 (25.0%)

**Figure 3:**
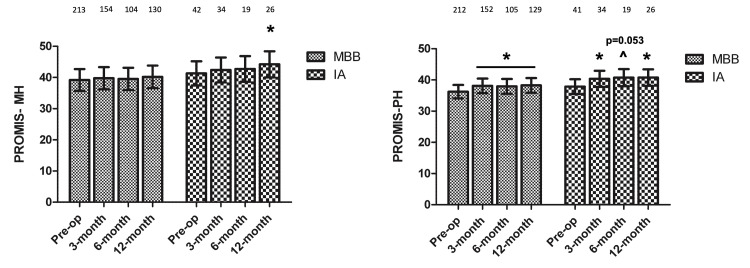
PROMIS-MH and PROMIS-PH scores before and after injection * = p<0.05; ^ = p trending IA: Intra-articular; MBB: Medial Branch Block; PROMIS: Patient-Reported Outcomes Measurement Information System; MH: Mental health; PH: Physical health

Regarding the PROMIS-MH score, the only statistically significant score change from baseline was observed at the one-year follow-up in the IA injection group, with improvement from baseline of 41.32 (95%CI 36.65-46.00) to 44.21 (95%CI 36.25-45.99). Conversely, statistically significant changes in the PROMIS-PH score were observed at the three-month, six-month, and one-year follow-ups in the IA injection group (average improvements of 2.48, 2.86, and 2.89, respectively, from baseline of 37.86 (95%CI 34.71-41.02)) and at three-month and one-year follow-ups in the MBB group (improvements of 1.89 and 2.04, respectively, from 36.22 (95%CI 33.21-39.22)) (Figure 3). The interaction between group and time point was not significant (P = 0.236) for PROMIS-MH and PROMIS-PH scores (P = 0.494), indicating that the trajectory of scores across time did not differ significantly between the two groups.

Finally, the mean PHQ-9 scores improved from a baseline of 8.48 (95%CI 5.65-11.32) in the IA injection group at both six-month and one-year follow-up by -2.05 and -2.75, respectively. In the MBB group, the mean improvement of PHQ-9 score was statistically significant only at the three-month follow-up, with a -1.66 improvement from the baseline of 10.02 (95%CI 7.32-12.72).

**Figure 4:**
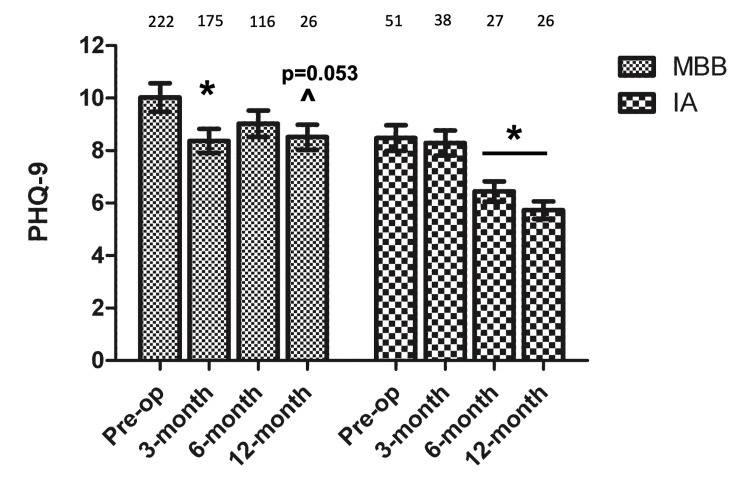
PHQ-9 scores before and after injection * = p<0.05; ^ = p trending IA: Intra-articular; MBB: Medial Branch Block; PHQ-9: Patient Health Questionnaire-9

The interaction between the treatment groups and time points was significant (p=0.016), which indicates that the two groups have different trajectories of PHQ-9 scores across time points. In the IA injection group, PHQ-9 scores were significantly lower at six-month and one-year follow-ups, while in the MBB group, PHQ-9 scores were significantly lower at three-month follow-up (Figure 4).

## Discussion

In the present study, we have examined the potential long-term relief of chronic LBP provided by lumbar MBB or IA injections with steroids using multiple quality-of-life measures. Our results demonstrate significant improvement in primary and secondary endpoints compared to pre-injection highlighting the efficacy of MBB and IA injection as potential therapeutic interventions. NRS scores of the MBB and IA injection groups demonstrated statistically and clinically significant improvement for one year. In patients who followed up, the frequency and percentage of patients who improved by MCID demonstrated a strong response (greater than 85% improvement) to either MBB or IA injection at three-month, six-month, and one-year follow-ups. Mean PROMIS-PH scores significantly decreased in the IA injection group at three-month, six-month, and one-year follow-ups, while statistically significant decreases were observed in the MBB group at three-month and 12-month follow-ups. Finally, the PHQ-9 mean scores were significantly lower for the IA group at six-month and one-year follow-ups and for the MBB group at the three-month follow-up.

Our results differ from those of previous studies that described MBB and IA injection as having ineffective therapeutic value [28]. The currently available research has yielded conflicting results, and societal guidelines do not support the use of therapeutic injections. Due to the uncertainty in the use of these procedures for therapeutic purposes, policy changes have recently been enacted which limited their coverage. Our findings indicate that MBB and IA injections can provide effective symptomatic management of chronic LBP over the long term, suggesting that these diagnostic modalities may have intrinsic therapeutic benefits.

Several studies have investigated the effectiveness of MBBs for facetogenic pain. A systematic review and meta-analysis concluded that MBBs with a combination of anesthetic and steroid were more effective than anesthetic alone for short-term pain relief (up to two weeks), but did not provide significant long-term pain relief (beyond three months) compared to anesthetic alone [17]. The study also reported that the use of MBBs did not result in significant improvements in functional outcomes or quality of life. Further, MBBs with a combination of anesthetic and steroid provided short-term pain relief, but while the evidence for long-term pain relief was limited, it is suggested that MBBs may be effective as part of a multimodal treatment approach for facetogenic pain [15]. Overall, the available evidence suggests that lumbar MBBs with a combination of anesthetic and steroid medication can provide at least short-term pain relief for facetogenic pain.

Similarly, results have been mixed regarding the effectiveness of IA injection for LBP suspected to be facetogenic or when diagnosis is suggested by a diagnostic anesthetic injection first. Additionally, even with an established diagnosis of LFJ pain (i.e., a positive IA diagnostic block), an IA injection of steroids was found to be no more effective than a normal saline injection [5,29-32]. In more recent research, IA injections have been shown to be ineffective in reducing the need for or the time to radiofrequency ablation of the medial branches in patients with dual MBB-confirmed LFJ pain [11].

Current 2020 North American Spine Society (NASS) LBP guidelines state that there is insufficient evidence to recommend for or against the use of steroid injections for suspected facet-mediated pain (Grade 1 Recommendation) and that there is not sufficient evidence for or against the use of 50% reduction in pain following MBB for diagnosis of facet joint pain (Grade 1 Recommendation) [30]. According to the guidelines, if therapeutic IA injections are used, they should be used in patients whose initial injections resulted in >50% pain relief for at least three months and should not be repeated more than three times annually. Our study data support the effectiveness of therapeutic MBBs and IA injections in a real-world scenario where patients have been referred for these procedures.

Limitations of the study

There are several limitations to this study, primarily due to the retrospective design. The intervention was performed by a singular physician at a large tertiary care center. This limits generalizability to other clinicians performing the procedure; however, this does reduce the variability of interventional technique. Additionally, NRS required direct patient inquiry and documentation in the EMR, whereas other PROs relied on patients fully completing survey data during their visit. The lack of standardized follow-up contributed to attrition at later time points and introduced potential confounding and follow-up bias. Patients who experienced improvement were more likely to return to the clinic rather than seek care elsewhere, while those with satisfactory symptom relief may not have followed up at all. Further, some patients returned to the clinic years later for unrelated symptoms, reporting complete relief of back or buttock pain following fluoroscopically guided MBB or LFJ injection.

Patients who did well may have returned to the clinic instead of following up with a new provider or may have not followed up at all if their pain had improved. We also report the frequency and percent of patients who improved by MCID for NRS, as well as those who experienced at least 50% improvement at each time point (Table 3). These response rates may reflect accurate patient selection (ie. LFJ as the primary pain generator) and contribute to the effects reported in the current study. However, some patients may have pain generators other than the LFJ; thus, diagnostic injection complicates the interpretation of results as patients with another etiology of pain likely would have different responses to treatment. Our diverging findings from prior clinical trials/studies may be attributed to several factors, such as improved approach techniques and patient selection resulting in greater accuracy of interventions [7]. Unfortunately, we were not able to quantify the amount of physical therapy patients performed during the study period. An adequately powered RCT with proper patient selection may help address these concerns. The authors of the current study note that the goal of the study was not to directly compare the two groups as it was not statistically feasible, given the difference in frequency between the groups.

It should also be noted that we analyzed patients by the type of their first injection, and only data from their first injection were used in analyses. Future studies evaluating the number of repeat injections over a given timeframe would be clinically important to evaluate. Our study found that of all patients in our database, a large portion did not undergo surgical intervention. More in-depth analysis of surgery rates would be clinically valuable and should be addressed in future RCTs.

Strengths of the study

Notable strengths of the study include treatment and evaluation by a single provider and institution, limiting variability in response, consistent patient selection, and technique. Further, our follow-up period represents a period longer than what is expected for corticosteroid response. Therefore, a sustained benefit may relate to other factors or management such as physical therapy, psychological support, use of oral analgesics, and disruption of the pain facilitation cycle, all of which are difficult to account for in a retrospective study. Following this work with randomized control trials (RCTs) comparing the current standard of MBB and IA injection followed by RFN treatment compared to injections alone will further elucidate the therapeutic potential of these modalities.

The primary advantage of this study is that it explores the therapeutic potential of MBB and IA injection, beyond diagnostics alone. Performing these procedures without RFN, as in the past, could change the paradigm of chronic back pain management. While results were more robust for the improvement of pain, some patients perceive benefits in their function, depression, and quality of life. Despite these promising conclusions, the results must be weighed against the current literature and limitations of the study, specifically the retrospective design and confounding variables. This warrants further prospective, multi-institutional investigation to better understand the therapeutic effectiveness of these interventions.

## Conclusions

Use of MBB and IA injection have been used primarily as diagnostic predictors for radiofrequency ablation success in chronic LBP. Based on the patients who maintained follow-up, greater than 85% continued to experience 50% pain relief at six months in our cohort. Therapeutic MBB and IA injection may be particularly beneficial for patients where RFN is contraindicated. These findings suggest a “real-life” clinical scenario where MBB and IA injection may have therapeutic benefits for facet-mediated low back pain beyond their current use and warrants further investigation.
